# Sensitive CometChip assay for screening potentially carcinogenic DNA adducts by trapping DNA repair intermediates

**DOI:** 10.1093/nar/gkz1077

**Published:** 2019-12-11

**Authors:** Le P Ngo, Norah A Owiti, Carol Swartz, John Winters, Yang Su, Jing Ge, Aoli Xiong, Jongyoon Han, Leslie Recio, Leona D Samson, Bevin P Engelward

**Affiliations:** 1 Department of Biological Engineering, Massachusetts Institute of Technology, Cambridge, MA 02139, USA; 2 Toxicology Program, Integrated Laboratory Systems, Inc., Research Triangle Park, NC 27560, USA; 3 Department of Biology, Massachusetts Institute of Technology, Cambridge, MA 02139, USA; 4 BioSystems and Micromechanics (BioSyM) IRG, Singapore-MIT Alliance for Research and Technology, 138602 Singapore; 5 Department of Electrical Engineering, Massachusetts Institute of Technology, Cambridge, MA 02139, USA

## Abstract

Genotoxicity testing is critical for predicting adverse effects of pharmaceutical, industrial, and environmental chemicals. The alkaline comet assay is an established method for detecting DNA strand breaks, however, the assay does not detect potentially carcinogenic bulky adducts that can arise when metabolic enzymes convert pro-carcinogens into a highly DNA reactive products. To overcome this, we use DNA synthesis inhibitors (hydroxyurea and 1-β-d-arabinofuranosyl cytosine) to trap single strand breaks that are formed during nucleotide excision repair, which primarily removes bulky lesions. In this way, comet-undetectable bulky lesions are converted into comet-detectable single strand breaks. Moreover, we use HepaRG™ cells to recapitulate *in vivo* metabolic capacity, and leverage the CometChip platform (a higher throughput more sensitive comet assay) to create the ‘HepaCometChip’, enabling the detection of bulky genotoxic lesions that are missed by current genotoxicity screens. The HepaCometChip thus provides a broadly effective approach for detection of bulky DNA adducts.

## INTRODUCTION

Injury to genetic material can lead to debilitating heritable diseases, cancer, neurodegeneration and accelerated aging (1–4). Therefore, regulatory agencies worldwide require that all pharmaceuticals be tested for their genotoxic potential (https://www.fda.gov/media/71980/download). In contrast, despite the fact that >2000 new chemicals are being produced by industry every year (https://ntp.niehs.nih.gov/annualreport/2017/2017annualreportdownloadpdf.pdf), the vast majority of these industrial chemicals have not been tested for their genotoxic potential. A major barrier to such testing is the need for a high throughput (HT) sensitive assay for DNA damage in mammalian cells (5). Although there have been recent advances in HT assays for genotoxicity (6), most of these technologies depend on indirect measures of DNA damage, such as phosphorylation of histones [e.g. γH2AX formation (7)] or gene induction [i.e. p53 activation (8,9)]. While there are several methods for direct detection of DNA damage (e.g. alkaline elution and mass spectrometry), these assays are laborious and low throughput. The alkaline comet assay is a promising platform as it detects single-strand breaks (SSBs; for a list of abbreviations, see Supplemental Table S1), abasic sites and other alkali sensitive sites. However, the assay has a critical blind spot, due to its inability to detect bulky DNA lesions, a class of lesions that are often carcinogenic (3,10,11). Here, we describe methods to overcome this limitation.

### The comet assay

The comet assay is an established method for detecting DNA strand breaks, and is based upon the underlying principle that fragmented DNA migrates more readily through an agarose matrix under electrophoresis compared to intact DNA. The comet assay works because nuclear DNA is normally highly supercoiled and thus does not readily migrate, while loops and fragments migrate more readily through the agarose matrix (12,13). The result is a comet-like shape, where the percent DNA in the comet tail is proportional to the levels of DNA strand breaks.

**Figure 1.**
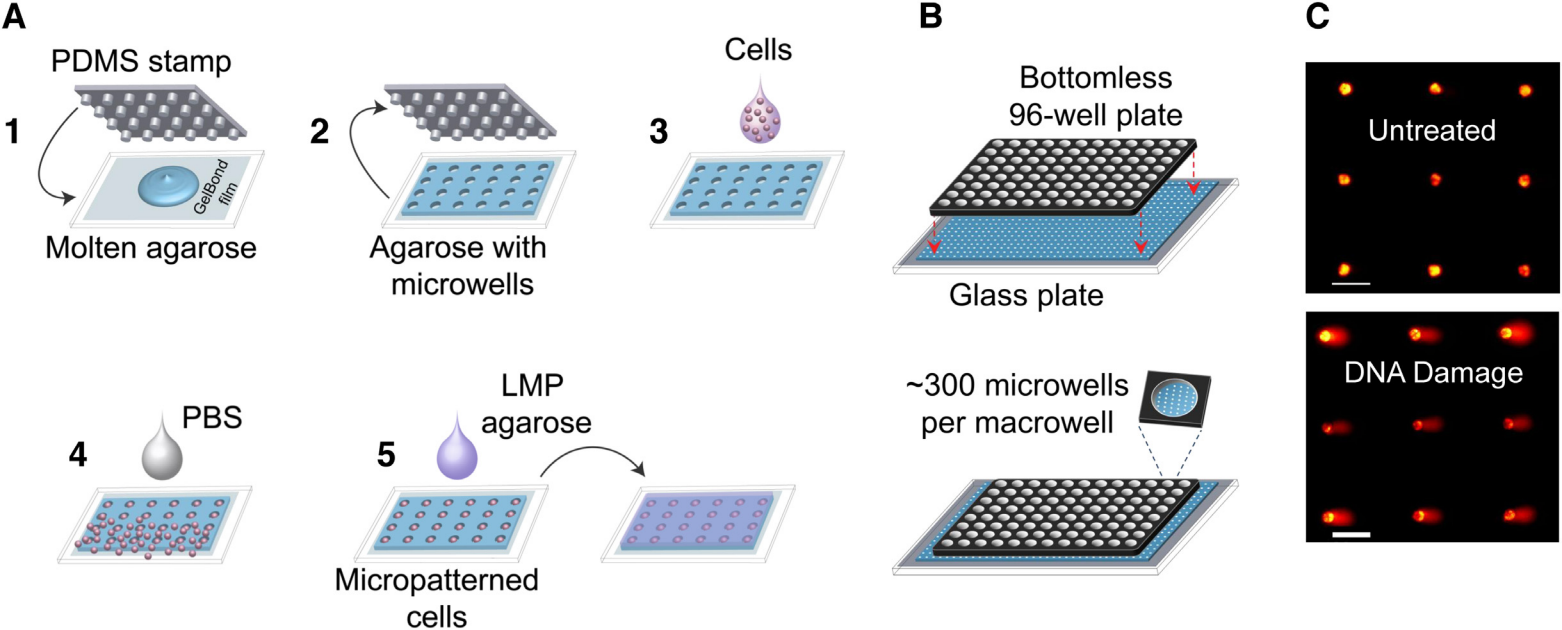
CometChip for high-throughput assessment of DNA damage. (**A**) CometChip fabrication. 1) A PDMS stamp with an array of micropegs is pressed into molten agarose. 2) Once the agarose gelates, the stamp is lifted to reveal an array of microwells (∼40–50 μm in both diameter and depth, spaced 240 μm from each other). 3) Cells in suspension are loaded directly into microwells via gravity. 4) Excess cells are washed off by shear force, revealing an array of micropatterned cells. 5) Low-melting point (LMP) agarose kept molten at ∼37°C is placed on top of the micropatterned cells and allowed to gelate by a brief incubation at 4°C (∼2 min). (**B**) Macrowells are formed by clamping a bottomless 96-well plate on top of a microwell array. The bottom surface of each macrowell contains ∼300 microwells. Macrowells can be used both to load multiple cell types at the same time and to perform parallel treatments. (**C**) Example fluorescent images of comets on alkaline CometChip. Images were taken at 4X magnification. Each image can capture ∼60–100 comet images. Upper: untreated TK6 cells yield comets with little to no tail. Lower: comets from TK6 cells treated with a high dose of a DNA damaging agent (50 μM H_2_O_2_) have visibly large tails. Scale bars = 100 μm.

While the comet assay is relatively simple and sensitive, it is low-throughput, it has poor reproducibility, and the imaging and analysis methods are laborious. To overcome these limitations, the CometChip was previously developed (14,15). The basis for the CometChip is an agarose microwell array. Briefly, cells are loaded into microwells by gravity, and excess cells are removed by shear force (Figure 1). By creating a mammalian cell microarray, overlapping comets are prevented, and the comets lie on a shared focal plane. As a result, it is possible to capture multiple comets (>50) in a single image rather than imaging each comet individually as is done for the traditional comet assay. With automated image analysis and reduced experimental noise, the CometChip provides >1000-fold improvement in throughput, increased robustness and increased sensitivity (14–18).

The comet assay can be performed using either neutral or alkaline conditions. Under alkaline conditions (pH > 13), SSBs release superhelical tension, enabling migration of DNA loops. Alkaline conditions also lead to SSBs at abasic sites and other alkali sensitive sites, which contribute to DNA migration. While broadly useful, alkaline comet conditions suffer from a major shortcoming, which is that the assay can only detect strand breaks that directly impact DNA migration and not base modifications or bulky DNA adducts. This is a significant limitation because many environmental carcinogens cause bulky DNA base adducts (3,10,11). Unrepaired adducts can block replication and transcription, which contributes to cell-cycle arrest, mutations, and cell death (19), ultimately contributing to carcinogenesis (20–24). In fact, high levels of bulky DNA adducts correlate with an increased risk of cancer in humans (25,26). Although the traditional alkaline comet assay does not detect DNA base lesions directly, they can be detected indirectly when acted upon by repair enzymes (27). For example, base excision repair (BER) enzymes remove damaged bases, cleave the backbone, synthesize across the gap, and ligate the DNA. As such, damaged bases lead to SSBs as requisite DNA repair intermediates, and these intermediates can be detected using the comet assay. Importantly, enzymes in the BER pathway can act independently, such that the subsequent SSB resolution steps are rate-limiting once the damaged base has been removed (28–30), leading to an accumulation of SSB intermediates that are detectable using the comet assay (27).

**Figure 2.**
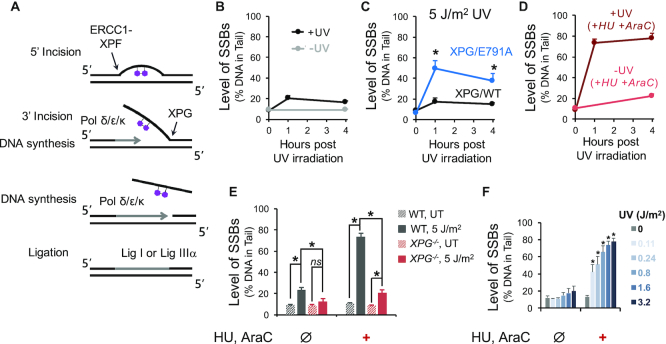
Analysis of SSBs by the alkaline CometChip as a measure of UV-induced lesions. Cells were pre-incubated with 10 mM GSH for 40 min at 37°C before UV irradiation and kept in the presence of 10 mM GSH for subsequent incubation following UV exposure. (**A**) Simplified schematic of NER of a UV-induced pyrimidine dimer. (**B**) Comparison of SSB levels in human skin fibroblast cell line (XPG/WT) between untreated cells (−UV) and cells irradiated with 5 J/m^2^ UV-C (+UV). (**C**) SSB levels in XPG/WT and XPG/E791A cells up to four hours following 5 J/m^2^ UV-C exposure. *P < 0.05, two-way ANOVA with post hoc analysis by Bonferroni test (between XPG/WT and XPG/E791A at each time point). (**D**) SSBs in XPG/WT cells incubated with DNA repair synthesis inhibitors, HU and AraC. Cells were pre-incubated with 1 mM HU, and 10 μM AraC for 40 min at 37°C, irradiated with 5 J/m^2^ UV-C (dark red line), and then incubated with the same HU and AraC concentrations for up to 4 h after exposure. Untreated control cells were kept in the same HU and AraC conditions (light red line). (**E**) Contribution of NER SSB intermediates to detected SSBs. XPG/WT and XPG-deficient cells were exposed to 5 J/m^2^ UV-C and allowed to repair for one hour following irradiation. Cells were either incubated with the repair synthesis inhibitors (1 mM HU, 10 μM AraC) for 40 min prior to UV irradiation and one hour of repair after exposure (+) or were incubated in regular medium without the inhibitors (Ø). ns: not statistically significant, *P < 0.05 (Student's *t*-test, two-tailed, paired). (**F**) HU/AraC approach reveals dose-response to UV exposure. TK6 cells were irradiated with indicated doses of UV-C and analyzed for SSBs 1 h following exposure. Cells were either incubated with the repair synthesis inhibitors (1 mM HU, 10 μM AraC) for 40 min prior to UV irradiation and 1 h of repair after exposure (+) or were incubated in regular medium without the inhibitors (Ø). *P < 0.05, one-way ANOVA with post hoc analysis by Dunnett's multiple comparison test (between each UV dose and the untreated control). *n*$ \ge$ 3. Error bars are standard error of the mean.

While many base lesions are repaired by BER, bulky lesions are repaired primarily by nucleotide excision repair (NER) (10,11,31,32) (Figure 2A). The NER pathway is highly coordinated in both prokaryotes and eukaryotes (31,33). In eukaryotes, the NER pathway operates by assembling more than a dozen different proteins prior to commencing repair (34). As such, once repair is initiated, the process is extremely efficient, thus minimizing the presence of SSB intermediates (35). Specifically, NER involves two major steps: endonucleolytic cleavage 5′ to the adduct, repair synthesis and endonucleolytic cleavage 3′ to the adduct (11,35), which together result in removal of an oligonucleotide containing the offending lesion. The incision 5′ to the damage site is made by the ERCC1-XPF endonuclease and is followed by recruitment of DNA polymerases and initiation of DNA repair synthesis. Repair synthesis creates a flap 3′ to the original lesion, which is then cleaved by the structure-specific endonuclease XPG, prior to completion of repair synthesis and ligation (36,37). NER SSB intermediates can be detected using the alkaline comet assay (38–40), but the required preassembly of the repair complex means that the signal is very weak due to the ephemeral nature of the NER SSB intermediates.

### Detecting bulky lesions using CometChip

Here, we exploit methods for inhibiting repair synthesis as a means for prolonging the presence of NER SSB intermediates. Specifically, our approach is to use hydroxyurea (HU) and 1-β-d-arabinofuranosyl cytosine (AraC), which inhibit NER (41–44). HU depletes the deoxyribonucleotide triphosphate (dNTP) pool by inhibiting the activity of ribonucleotide reductase (45–49). AraC is a deoxycytidine structural analog (50), which can be incorporated into DNA (51–54), inhibiting DNA elongation by DNA polymerases and causing early chain termination (51,55–58). Despite the potential utility of HU/AraC as a means for making the comet assay more sensitive (39,59–64), little has been done to leverage and/or validate the utility of this approach. Developing an effective CometChip-based assay for detecting bulky lesions has the potential to give rise to a valuable tool for rapid detection of carcinogenic bulky lesions. Therefore, leveraging the high-throughput nature of CometChip, we set out to develop a rapid and sensitive assay for bulky lesions by using HU/AraC to trap NER repair intermediates and reveal SSBs generated during NER.

While bulky lesions can be created by direct chemical reactions with DNA, some chemicals lead indirectly to the creation of bulky lesions. For example, polycyclic aromatic hydrocarbons (PAHs), such as benzo[a]pyrene (B[a]P) and aflatoxin B_1_ (AFB_1_), do not react with DNA unless they are rendered reactive by metabolic enzymes (a.k.a. metabolic activation). In the human body, foreign substances (xenobiotics) are extensively metabolized, mainly by hepatocytes in the liver (65). Metabolism can convert a lipophilic molecule into a soluble molecule, thus aiding in its excretion. In some cases, metabolism can lead to formation of highly reactive and toxic intermediates. In fact, liver toxicity is a major problem in drug development and for public health. Drug-induced liver injury (DILI) remains a common cause for drug withdrawal from the market and is the most common cause of acute liver failure and death. In addition, the liver remains the most frequent target organ in rodents for >500 environmental chemicals tested as part of the EPA Integrated Risk Information System (IRIS) (www.epa.gov/iris) (27). Therefore, in order for chemical toxicity assessment to be physiologically relevant, it is essential to have a testing system that can provide biologically relevant levels of xenobiotic metabolism.

The biotransformation process of xenobiotics includes oxidation/reduction of parent chemicals, increasing their hydrophilicity by adding polar groups (such as hydroxyl or amine) and endogenous polar compounds, making them more soluble and thus more easily cleared via the bloodstream (65,66). The cytochrome P450 enzymes (CYP450s), or microsomal mixed-function mono-oxygenases localized to the endoplasmic reticulum, account for ∼75% of all phase I enzymes (66) and are involved in ∼95% of oxidative biotransformation (65). Phase I enzyme biotransformation creates functional groups that are then substrates for conjugation to water soluble molecules (e.g. glucuronic acid, sulfate or the tripeptide glutathione) by Phase II enzymes, greatly increasing the polarity of the metabolites from phase I and suitable for excretion (65). Given that oxidation products of CYP450s can become DNA reactive, assessment of chemical genotoxicity needs to take into consideration the genotoxic potential of both the parent chemicals and their metabolites. As an example, the DNA damaging effects of B[a]P, a major public health hazard that may lead to hundreds of thousands of cancer cases each year, would be entirely missed in laboratory tests were it not for metabolic activation. HepaRG cells can undergo extensive differentiation, exhibiting hepatocyte-like morphology as well as displaying substantial liver-specific functions. Here, we have developed the HepaCometChip, an enhanced CometChip (15) platform for genotoxicity screening, by incorporating the use of two DNA repair synthesis inhibitors, HU and AraC (to enable persistence of NER SSB intermediates) and HepaRG™ cells (to enable metabolic activation).

The carcinogens AFB_1_ and B[a]P are used as model DNA damaging agents that require metabolic activation to be reactive with DNA. The mycotoxin AFB_1_ is produced by the molds *Aspergillus flavus* and *A. parasiticus*, which are frequent contaminants in peanuts and maize in certain regions of the world (67). Several CYP450 enzymes (68), such as CYP3A4 and CYP1A2, are known to oxidize AFB_1_, producing a number of metabolites (69–72). The highly mutagenic metabolite, AFB_1_-exo-8,9-epoxide, readily reacts with the N7 position of guanine to form DNA adducts. The most ubiquitous are AFB_1_-N7-dG and AFB_1_-Fapy-dG (21,22). In addition to AFB_1_, we also elected to study B[a]P, another human carcinogen (http://monographs.iarc.fr/ENG/Classification/latest_classif.php). Common routes of exposure include breathing in fuel exhaust, cigarette smoke, and burning wood smoke, or consuming charred meat or other types of charred food (24). B[a]P, like many other PAHs, is an inducer of the CYP1 family (CYP1A1, CYP1A2, and CYP1B1) (73). B[a]P is also metabolically activated by the CYP1 family. The most genotoxic metabolite is the diolepoxide (+)-anti-B[a]*P*-7,8-diol-9,10-epoxide (BPDE), which covalently binds to the exocyclic *N*^2^ of guanine. Both AFB_1_-N7-dG and BPDE-DNA lesions highly distort the double helix and are excised by NER (24,74).

In this study, we report the development of the HepaCometChip, a HT screening platform that is highly effective for detecting repair intermediates of bulky lesions. We leveraged the existing high-throughput CometChip platform (15,17) and knowledge of how bulky lesions are normally repaired, in order to trap repair intermediates and use these intermediates as indicators of the presence of bulky lesions (10,11,32). In addition, we exploited metabolically competent human hepatic cells to account for metabolic processes that can convert non-reactive molecules into DNA-reactive molecules that form bulky lesions (65,66). Further, using specific CYP450 inhibitors, we validated that the detected SSBs are primarily dependent upon metabolic activation of B[a]P and AFB_1_. We also showed that inhibition of NER initiation results in a reduction in SSB levels, indicating that NER intermediates contribute significantly to SSBs detected using the HepaCometChip. Furthermore, we demonstrated that the platform has superior sensitivity compared to the conventional alkaline comet procedure by performing a small screen of nine known *in vivo* genotoxic agents. Taken together, we have leveraged the HT advantage of CometChip, the enhanced comet sensitivity in the presence of HU/AraC, and the metabolic capacity of HepaRG™ cells to develop the HepaCometChip screening platform for single strand breaks induced by the metabolism of xenobiotics. This platform provides an unprecedented rapid and sensitive tool to help overcome vital limitations in current genotoxicity testing performed by regulatory agencies, public health researchers, and pharmaceutical companies.

## MATERIALS AND METHODS

### Chemicals

Sodium bicarbonate solution (7.5% NaHCO_3_, S8761), dimethyl sulfoxide (DMSO, D2650), reduced l-glutathione (GSH, G6013), hydroxyurea (HU, H8627) and cytosine arabinoside (AraC, C1768) were obtained from MilliporeSigma, St Louis, MO. GSH solution (10 mM) was prepared by dissolving GSH powder in warm culture medium and used within 30 min of preparation. Stock solutions of 1000× HU (1 M) and 1000× AraC (10 mM) were prepared by dissolving crystal HU and AraC in cell culture grade water and stored at −20°C. Other chemicals (Supplementary Table S2) were purchased in powder form from MilliporeSigma, St Louis, MO and dissolved to prepare stock solutions that were stored at −20°C.

### Cell culture

Dulbecco's phosphate-buffered saline (PBS), high-glucose Dulbecco's modified Eagle's medium (DMEM, high glucose, 11965092), RMPI-1640 with GlutaMAX™ (61870), 200 mM l-glutamine (A2916801), 10 000 U/ml Pen-Strep (15140), 100× GlutaMAX™ supplement (35050061), William's E Medium (WEM, A1217601), HepaRG™ Thaw, Plate, & General Purpose Medium Supplement (HPRG670), HepaRG™ Maintenance/Metabolism Medium Supplement (HPRG620), 0.25% Trypsin-EDTA with phenol red (25200), and 96-well plate coated with collagen I (A1142803) were purchased from ThermoFisher Scientific, Waltham, MA. Fetal bovine serum was obtained from Atlanta Biologicals, Inc., Flowery Branch, GA.

All cells were cultured in an incubator set at 37°C with 5% CO_2_. TK6 (75,76), a human B-lymphoblastoid cell line, was a gift from W.G. Thilly (Department of Biological Engineering, Massachusetts Institute of Technology). TK6 cells were cultured in RPMI 1640 medium with GlutaMAX™ supplemented with 100 U/ml Pen-Strep. The XPG cell lines were gifts from O.D. Scharer (Institute for Basic Science, Center for Genomic Integrity, Ulsan, Korea). These include XPG-deficient, XPG/WT, and XPG/E791A. The XPG-deficient cell line was obtained from SV40-transformation of the primary human skin fibroblasts from patient XPCS1RO (77). XPG/WT and XPG/E791A cells were obtained from the stable transfection of the lentiviral vector containing XPG WT cDNA or XPG-E791A cDNA in the XPG-deficient cell line (35). The XPG cell lines were cultured as previously described (35,77).

HepG2 (ATCC^®^ HB-8065™), an immortalized cell line derived from human hepatocellular carcinoma, was obtained from the American Type Culture Collection (Manassas, VA). HepG2 cells were cultured in high-glucose DMEM supplemented with 10% FBS, 1× GlutaMAX™, and 100 U/ml Pen-Strep. For chemical exposures, exponentially growing cells were plated in a tissue cultured treated 96-well plate two days before treatment.

Cryopreserved HepaRG™ (HPRGC10), a terminally differentiated hepatic cell line, was purchased from ThermoFisher Scientific (Waltham, MA). HepaRG™ was thawed and cultured according to manufacturer's instructions. Briefly, the general purpose working medium was WEM supplemented with 1X GlutaMAX™ and 1X HepaRG™ Thaw, Plate, & General Purpose Medium Supplement. The metabolism working medium was WEM supplemented with 1X GlutaMAX™ and HepaRG™ Maintenance/Metabolism Medium Supplement. HepaRG™ cells were thawed in the general purpose working medium and plated in a 96-well plate coated with collagen I at 100 000 cells/well. One day after plating, the general purpose working medium was changed to the metabolism working medium. The metabolism working medium was renewed on day 4 and day 6 after plating. On day 7, the cells were treated in the metabolism working medium.

To obtain a cell suspension for the XPG cell lines, HepG2 and HepaRG, the monolayer culture was incubated with 0.25% Trypsin–EDTA at 37°C. For XPG cell lines, the incubation time was 1–2 min. For HepG2 and HepaRG, the incubation time was 5–10 min. Detached cells were then suspended in complete working media. Cell viability and cell number were analyzed using an automated Trypan Blue exclusion system [Vi-CELL™ cell counter (Beckman Coulter Life Sciences, Brea, CA, USA)].

### CometChip fabrication

Sylgar™ 184 silicone elastomer kit (102092-312) and bottomless 96-well plates (82050-714) were purchased from VWR, Radnor, PA. GelBond^®^ Film (53761) was obtained from Lonza, Portsmouth, NH. UltraPure™ agarose (16500100) and UltraPure™ low melting point agarose (16520100) were purchased from ThermoFisher Scientific, Waltham, MA. The microwells were fabricated as described previously (14–16,78). Briefly, 1% (w/v) agarose solution in PBS was prepared. A polydimethylsiloxane (PDMS) stamp with an array of micropegs was fabricated using the Sylgar™ 184 kit as described previously (15). The stamp was pressed into the molten agarose solution on top of the hydrophilic side of a sheet of GelBond® film. The agarose was allowed to gelate at room temperature for ∼15 min. The stamp was removed to reveal an array of microwells with ∼40–50 μm in both diameter and depth. The microwells were spaced ∼240 μm apart. A bottomless 96-well plate was pressed on top of the agarose chip to form 96 macrowells, each with an array of ∼300 microwells at its base.

To load cells into microwells, ∼2000–200 000 cells in suspension were placed into each macrowell, and the chip was incubated at 37°C in the presence of 5% CO_2_ for 15 min. Cell were loaded into microwells by gravity, and excess cells were then washed off with PBS by shear force. The chip was covered with a layer of overlay agarose (1% w/v low-melting point agarose solution in PBS, kept molten at 43°C until use). For complete gelation of the overlay agarose, the chip was kept at room temperature for two minutes followed by 2 min at 4°C.

### Trypan Blue exclusion test for cell viability

HepaRG™ cell viability was determined using an automated Trypan Blue exclusion system [Vi-CELL™ cell counter (Beckman Coulter Life Sciences, Brea, CA, USA)]. HepaRG™ cells were incubated with various doses of HU/AraC in triplicates for 24 h at 37°C in the presence of 5% CO_2_. A vehicle control (1% DMSO) was included. The number of viable cells was recorded for each dose of HU/AraC and % control viability calculated.

### Alkaline comet assay

The alkaline comet assay was performed as previously (15,79). Sodium chloride (NaCl, 7581), disodium EDTA (Na_2_EDTA, 4931), and sodium hydroxide pellets (NaOH, 7708) were purchased from VWR, Radnor, PA. Trizma^®^ base (T1503), Trizma^®^ HCl (T5941) and Triton X-100 (X-100) were obtained from MilliporeSigma, St. Louis, MO. 10 000× SYBR™ Gold nucleic acid gel stain was obtained from ThermoFisher Scientific, Waltham, MA.

The alkaline lysis buffer (pH ∼ 10) was a solution of 2.5 M NaCl, 100 mM Na_2_EDTA, 10 mM Trizma® base, and 1% (v/v) Triton X-100 dissolved in deionized H_2_O (dI H_2_O). The alkaline unwinding buffer (pH ∼ 13.5) was prepared by diluting NaOH and Na_2_EDTA stock solutions in dI H_2_O to final concentrations of 0.3 M and 1 mM, respectively. The neutralization buffer (pH ∼ 7.5) was prepared by dissolving Trizma® HCl in distilled H_2_O to a final concentration of 0.4 M.

Cells encapsulated in CometChip were lysed in the alkaline lysis buffer overnight at 4°C. The nuclei were unwound in the alkaline unwinding buffer for 40 min at 4°C, and the DNA was electrophoresed in the same buffer at the same temperature for 30 min at 1 V/cm and ∼300 mA. The CometChip was then washed three times in neutralization buffer by submerging for 5 min each time.

The DNA on CometChip was stained for 15 min at room temperature with 1× of SYBR™ Gold diluted in PBS, protected from light. Fluorescent images of the comets were captured at 40× magnification using an epifluorescence microscope (Nikon Eclipse 80i, Nikon Instruments, Inc., Melville, NY, USA) with a 480 nm excitation filter. Image acquisition was achieved by automatic scanning using a motorized XY stage. Comet images were automatically analyzed using Guicometanalyzer, a custom software developed in MATLAB (The MathWorks Inc., Natick, MA, USA) as previously described (15). For each condition, 100 comets or more were analysed. Outputs from Guicometanalyzer were processed and imported to a spreadsheet (Microsoft Excel, Microsoft Office Suite 2016) using Comet2Excel, an in-house software developed in Python (Python Software Foundation, Python version 2.7.10). Software is freely available upon request.

Liver perfusion and hepatocyte culture on CometChip. Gibco^®^ Antibiotic-Antimycotic (15240, 100×) was purchased from ThermoFisher Scientific, Waltham, MA. Insulin-transferrin-sodium selenite supplement (ITS) (11074547001), aprotinin (A3428), HEPES (H4034), dexamethasone (D4902), and Percoll^®^ (P4937) were obtained from MilliporeSigma, St. Louis, MO. Isolation medium was WEM supplemented with 1X GlutaMAX™, 1× Gibco^®^ Antibioti-Antimycotic, 10 μg/ml IST, 1 μg/ml aprotinin, 10 mM HEPES, 0.1 μM dexamethasone and 10% FBS. Maintenance medium was the same as isolation medium, but without 10% FBS.

Primary mouse hepatocytes were obtained from 10 to 14 weeks old C57Bl6 mice using a standard two-step collagenase liver perfusion procedure with minor changes (80,81). The isolated cells were suspended in the isolation medium and were enriched for viable hepatocytes by centrifugation using a 45% Percoll^®^ solution. Cell viability and cell number were analyzed using an automated Trypan Blue exclusion system [Vi-CELL™ cell counter (Beckman Coulter Life Sciences, Brea, CA)]. The perfusion procedure yielded ∼20–50 million cells per liver and ∼80–90% cell viability.

Microwell array in agarose chip was fabricated as described above with some changes. Specifically, the 1% w/v agarose solution and the overlay agarose solution were supplemented with 2× Gibco^®^ Antibiotic–Antimycotic. Hepatocytes in suspension were loaded into microwells by incubating at 37°C for a maximum of 10 minutes. After the agarose overlay step, cells were incubated in isolation medium (50 μl/macrowell) at 37°C in the presence of 5% CO_2_. After 4 h, isolation medium was exchanged for maintenance medium, and cells were incubated overnight at 37°C in the presence of 5% CO_2_. After the overnight incubation, chemical treatments were performed in the maintenance medium (50 μl/macrowell).

### HU/AraC treatments

HepaRG^TM^ were treated with varying concentrations of HU/AraC in a 96-well plate for 24 h at 37°C in the presence of 5% CO_2._ The treated cells were embedded onto a CometChip and the level of damage induced by HU/AraC analyzed by Alkaline comet assay. For this experiment, the comets were analysed using Trevigen^®^ Comet Analysis Software.

Ultraviolet (UV) light exposure. Prior to UV irradiation, cells embedded in CometChip were incubated for 40 min at 37°C in working medium supplemented with 10 mM glutathione. Exposure to 254 nm UV light radiation (UVC) was administered via a handheld UV lamp that had a dose-rate of 14 J/m^2^/s at a distance of 7.6 cm (UVP 95001614, ThermoFisher Scientific, Waltham, MA, USA). The UV irradiation procedure was carried out in the dark at 4°C.

HU/AraC approach to query UV-induced bulky adducts. A combination of 1 mM HU and 10 μM AraC was used to inhibit NER repair synthesis. Before UV exposure, cells were pre-treated with HU/AraC prepared in working medium supplemented with 10 mM GSH for 40 min at 37°C. Following UV exposure, cells were incubated in working medium supplemented with 10 mM GSH and HU/AraC for 1 h and 4 h at 37°C, and analyzed by alkaline comet.

### AFB_1_ and B[a]P treatments

For each dose of the test compound, a 200× solution was prepared by diluting the stock solution (4 mM AFB_1_ or 20 mM B[a]P) in DMSO. A vehicle control condition was included by diluting DMSO in cell culture medium to get a final concentration of 0.5%. Cells were incubated with the test compound for 24 h at 37°C in the presence of 5% CO_2_. To reveal the level of DNA damage induced by the test compound, cells were also exposed to HU/AraC.

HU/AraC approach in chemical screen of nine known genotoxins. HepaRG™ were incubated with the test compound for 24 h at 37°C in the presence of 5% CO_2_. A vehicle control (1% DMSO) was included.

HU/AraC approach to test for genotoxicity of artesunate. HepG2 were incubated with the test compound for 24 h at 37°C in the presence of 5% CO_2_. A vehicle control (0.07% sodium bicarbonate) was included.

### Inhibition of AFB_1_ or B[a]P metabolic activation

5 μM KET or 25 μM ANF was added to culture medium at the start of the AFB_1_ or B[a]P treatment. The remaining steps were similar to the AFB_1_ and B[a]P treatments.

### Gamma radiation

Cells embedded in CometChip were placed on ice and irradiated with 0, 0.9, 1.8, 3.6, 5.4, 7.2 and 9 Gy of gamma rays from a Cs 137 radiation source with a dose rate of 0.9 Gy/min. The irradiated cells were submerged into lysis buffer overnight and the alkaline CometChip assay performed as described above. Standard curves with the numbers of SSBs induced at each radiation dose versus the change in % Tail DNA were generated and the slopes of the curves (SSBs induced/change in % tail DNA) used to estimate the number of SSBs induced at the highest concentrations of B[a]P and AFB_1_ for in each cell line.

### CellTiter-Glo^®^ assay (CTG^®^) for cell viability

The CTG^®^ luminescent cell viability assay kit (G7570) was obtained from Promega, Madison, WI. Cell viability after 24 h of chemical treatment was measured according to the manufacturer's instructions. Luminescent signals were recorded using a SpectraMax M2e microplate reader (Molecular Devices, Sunnyvale, CA, USA) at room temperature. The emission bracket was from 360 to 750 nm. Control wells with no cells were included to obtain background luminescence, which was then subtracted from the signal measured in the sample wells.

### CYP450-Glo™ assays for CYP3A4 and CYP1A2 activity

The CYP450-Glo™ CYP3A4 assay with luciferin-IPA (V9001) and the CYP450-Glo™ CYP1A2 assay with luciferin-1A2 (V8421) were purchased from Promega, Madison, WI. The activity levels of CYP3A4 and CYP1A2 in cells were measured according to the manufacturer's protocol for non-lytic cell-based assays. Luminescent signals were recorded using a SpectraMax M2e microplate reader (Molecular Devices, Sunnyvale, CA, USA). Control wells with no cells were included to obtain background luminescence. The net signal for each sample was obtained by subtracting the background luminescence value.

CYP3A4 or CYP1A2 activity per cell can be obtained by normalizing CYP450-Glo™ values with cell numbers. After a sample was analyzed for CYP3A4 or CYP1A2 activity, the CTG^®^ assay was performed to obtain an estimate of the number of viable cells in the sample. The result from CYP450-Glo™ was then divided by the CTG^®^ value to obtain the average CYP3A4 or CYP1A2 activity per cell.

## RESULTS

### Application of HU/AraC for sensitive detection of bulky DNA adducts using the comet assay

Many environmental carcinogens, such as ultraviolet light, PAHs, and heterocyclic amines, induce DNA lesions that are considered to be ‘bulky’ (10,11). Bulky adducts thermodynamically destabilize the double helix, which helps to enable NER recognition (11). After the NER machinery has been assembled at the site of the DNA lesion, incisions are made to excise a 22–30 nucleotide fragment containing the lesion (32). As a consequence, SSBs are requisite intermediates of the pathway, and these intermediates are, in principle, detectable using the alkaline comet assay. However, it has been reported that the frequency of NER associated SSBs is relatively low, even for high UV doses (82). Poor detection of NER intermediates is consistent with SSBs being transient and thus difficult to detect with the alkaline comet assay. A major goal was therefore to render SSBs longer-lived, making them easier to detect, thus enabling their use as a metric for the presence of bulky lesions.

To study NER-induced SSBs, we first performed the traditional alkaline comet procedure using CometChip (15,16,78) (Figure 1). In order to investigate the sensitivity of the CometChip to detect NER-induced SSBs at sites of bulky DNA lesions, we exposed immortalized human fibroblasts to UV-C, which induces mostly CPDs and 6–4PPs (32), known substrates of NER (Figure 2A). After exposure to UV-C, cells were allowed to repair for up to 4 h. As shown in Figure 2B, the level of DNA SSBs analyzed by the alkaline comet assay increases slightly following UV exposure, reaching a maximum of ∼20% tail DNA after 1 h. This level of damage is only marginally above the basal damage level (∼10% tail DNA) and well below the alkaline comet assay's saturation level (∼75% tail DNA), indicating that the assay is relatively insensitive to bulky adducts.

The levels of SSBs are a function of both break generation (incision), gap filling (synthesis) and ligation. Therefore, inhibiting gap filling or ligation can theoretically increase the level of SSBs, thereby improving the assay's sensitivity. To test the possibility that inhibition of ligation increases sensitivity of the assay, we exploited XPG/E791A mutant cells, for which NER intermediates are predicted to persist. Specifically, the E791A mutation renders the XPG enzyme catalytically inactive, but still allows assembly of the NER machinery (35,83). Because the 5′ incision by ERCC1-XPF is not affected (35), XPG/E791A cells are able to generate NER SSB intermediates. However, XPG-E791A is not able to cleave 3′ to the damage site, leading to a 5′ overhang and preventing ligation (35). Consistent with this model, we observed a significant increase in the level of SSBs following exposure to UV (Figure 2C), agreeing with previous studies (35,83,84).

Given that inhibition of NER completion via catalytic inactivation of XPG leads to increased NER intermediates, it follows that the sensitivity of the assay should similarly be increased via inhibition of the gap filling that precedes ligation. In fact, classic SSB detection methods, such as the alkaline sucrose sedimentation technique (43,64) and alkaline elution assay (85), employ the use of DNA repair synthesis inhibitors to increase the levels of SSBs resulting from initiation of NER at sites of UV-induced DNA damage. We adapted this technique by employing a combination of the DNA replication inhibitors HU and AraC for the alkaline CometChip assay, similar to previously described studies (38–40,86,87). We performed a Trypan Blue exclusion test to determine HU/AraC toxicity, and a Comet assay to determine level of DNA damage induced by HU/AraC. We chose a concentration of 1 mM HU and 10 μM AraC, for which there is at least 80% survival and no statistically significant change in DNA damage in HepaRG™ cells (Supplemental Figure S1). In the presence of HU/AraC, the level of accumulated SSBs reaches a steady state of ∼73–78% tail DNA after 1 h following UV exposure (Figure 2D), which is approximately four times higher than the control condition. Since ∼75% tail DNA is approximately the alkaline comet assay's saturation limit, it is possible that an even higher number of SSBs are generated.

### NER intermediates contribute to UV-induced SSBs detected by HU/AraC

To formally test the hypothesis that HU/AraC leads to accumulation of NER-driven SSBs, we evaluated the level of UV-induced SSBs in cells that are not able to perform NER. Complete lack of XPG prevents formation of the pre-incision complex, thus preventing incision (35). For WT cells, UV alone leads to a slight increase in SSBs as expected (Figure 2E). This level is significantly reduced in the absence of XPG. In the presence of HU/AraC, the level of SSBs is greatly increased in WT cells. In contrast, cells completely lacking XPG show only a slight increase in SSBs (Figure 2E), demonstrating that almost all of the SSBs detected using HU/AraC are NER intermediates.

To address the generalizability of the approach, we performed a dose-response experiment. TK6 cells were irradiated with various doses of UV-C and allowed to repair in the presence of HU/AraC for one hour after UV exposure (to maximize SSB accumulation). While control cells treated without HU/AraC show a trend but no significant increase in SSBs, cells treated with HU/AraC show a strong increase in SSBs in a dose-dependent fashion (Figure 2F). Furthermore, given that UV is a direct acting DNA damaging agent, cells with and without the ability to undergo metabolic activation are anticipated to respond similarly to UV, which is indeed the case (Supplemental Figure S2).

### Application of HU/AraC to detect DNA damage induced by metabolic activation of AFB_1_ and B[a]P

Given the importance of metabolism for converting non-genotoxic compounds into genotoxic agents, we set out to create a CometChip procedure that is compatible with endogenous biotransformation to form reactive metabolites. To study DNA damage in physiologically relevant metabolic conditions, we developed the HepaCometChip by combining the HU/AraC approach with metabolically competent human cells. We tested the efficacy of the method by treating HepaRG™ and HepG2 with the carcinogens AFB_1_ and B[a]P, both of which are known to become DNA reactive upon metabolic activation by CYP450s and to form bulky adducts recognized by NER. To control for metabolic activation, we included a negative control cell line, TK6, which does not express CYP450s (88).

**Figure 3.**
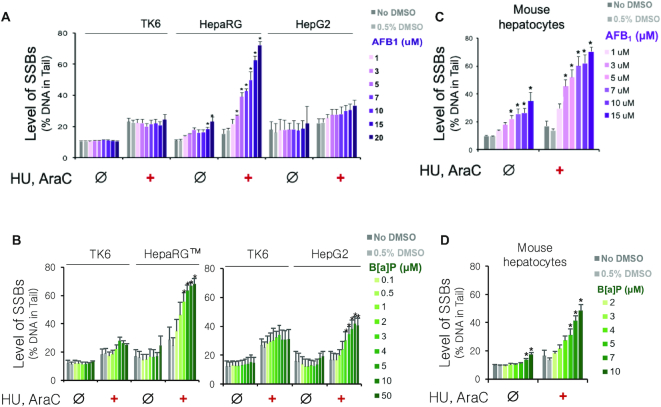
Application of HU/AraC approach on alkaline CometChip to detect DNA damage induced by metabolic activation of AFB_1_ and B[a]P. Cells were treated with either AFB_1_ or B[a]P in the absence (Ø) or presence (+) of 1 mM HU and 10 μM AraC for 24 h at 37°C and analyzed with the alkaline CometChip. (**A**) Dose-response to AFB_1_ in TK6, HepaRG™ (same-day treatment), and HepG2. All three cell lines were treated in parallel. (**B**) Dose-response to B[a]P in TK6, HepaRG™ (day-7 treatment, see Materials and Methods), and HepG2. HepaRG™ and HepG2 cells were treated on different days. TK6 was analyzed in parallel as a control for each treatment. *n*$ \ge$ 3. Error bars are standard errors of the means. (**C** and **D**) Dose-response to AFB_1_ (C) and B[a]P (D) in primary mouse hepatocytes. All data represent the average of six mice (C57Bl6, 10–14 weeks old). Error bars are standard error of the mean. *P < 0.05, one-way ANOVA with post hoc analysis by Dunnett's multiple comparison test [between treated dose and vehicle control (0.5% DMSO)].

We first looked at the levels of SSBs induced by AFB_1_. In HepaRG™ cells, there is a strong dose-response relationship to AFB_1_ treatment only in the presence of HU/AraC (Figure 3A). In contrast, the metabolically incompetent TK6 cells display no response to AFB_1_ treatment (Figure 3A), supporting the role of metabolic activation in SSB formation. It should be noted that HU/AraC leads to a slight increase in SSBs in the absence of AFB_1_, which is likely due to detection of spontaneous repair. Next, we looked at HepG2 cells, which are commonly used for studies that require metabolic activation. We found that in the presence of HU/AraC, there is a relatively small increase in SSBs induced by AFB_1_ compared to the dose-response observed in HepaRG™ cells. This result is consistent with the fact that HepG2 cells are known to express CYP450s at a much lower level compared to HepaRG™ cells (89–100). In fact, we measured the activity levels of two enzymes essential in metabolic activation of AFB_1_, CYP3A4 and CYP1A2, and found that HepG2 exhibits much lower levels of both of these CYP450s. Specifically, HepG2 shows >100-fold lower CYP3A4 activity (Supplemental Figure S3) and >10-fold lower CYP1A2 activity compared to HepaRG™ cells (Supplemental Figure S4).

Similar to AFB_1_ treatment, B[a]P also does not induce SSBs in the metabolically incompetent TK6 cells (88). In contrast, in the presence of HU/AraC, there is a dose-dependent increase of SSBs in both HepaRG™ and HepG2 cells (Figure 3B). These results are consistent with the fact that both of these cell lines are known to have an inducible CYP1 system, supporting the role of metabolic activation in B[a]P-induced DNA damage. In fact, the activity level of CYP1A2, one of the key metabolic enzymes of B[a]P, is induced in a dose-dependent manner by B[a]P in both HepaRG™ and HepG2 cells (Supplemental Figure S4). Importantly, HepaRG™ cells display overall higher levels of SSBs compared to HepG2 (Figure 3B) consistent with the observation that HepaRG™ cells express >10-fold higher CYP1A2 activity level compared to HepG2 cells (Supplemental Figure S4).

### Calibration results using gamma radiation

To provide more quantitative estimates for the number of SSBs (bulky adduct repair intermediates) detected by the alkaline CometChip assay, we performed a dose-response experiment using gamma radiation for which the strand breaks induced per Gy are known in order to obtain a standard curve for each cell line. TK6, HepG2 and HepaRG™ cells were irradiated with various doses of gamma radiation, and the levels of SSBs were assessed using the alkaline CometChip. We observed a dose-dependent increase in % Tail DNA (indicative of SSBs) in each cell line (Supplemental Figure S5A). Note that the dose response curve is distinct for each cell line, indicating that the same number of SSBs result in different levels of %tail DNA in different cell types. We then estimated the number of SSBs/cell induced by the highest concentrations of B[a]P and AFB_1_ in each cell line using calibration curves from the radiation data. To generate calibration curves, we first converted the radiation doses into the number of SSBs (induced by radiation) using the estimation that 1Gy of gamma radiation induces 1000 SSBs (101). Next, we calculated the induced % Tail DNA by correcting the background (non-radiated cells) in all the radiation treated results. The slope of the standard curve (SSBs induced/change in % Tail DNA) was then used to estimate the number of SSBs induced by B[a]P and AFB_1_ (Supplemental Figure S5B, and Table S3). The results show that the presence of HU/AraC reveals thousands of SSBs induced by B[a]P and AFB_1_ in HepaRG and HepG2 cells, indicative of thousands of bulky lesions that are missed without HU/AraC. Also, the correction for background damage resulted in negative numbers in a few cases where there is very little or no induced damage. The negative numbers are therefore an artefact of the calculation and are considered to reflect no change in DNA damage compared to background.

### Application of HU/AraC in primary mouse hepatocytes

Primary hepatocytes are the gold standard for metabolism studies (102). Therefore, we wanted to test the efficacy of HU/AraC in these cells. Mouse hepatocytes were isolated using a standard two-step collagenase liver perfusion (80,81). The cells were immediately loaded into CometChip microwells and allowed to recover overnight before AFB_1_ or B[a]P treatment. Unexpectedly, we observed that AFB_1_ induces SSBs that are detectable even in the absence of HU/AraC (Figure 3C). A potential reason is because AFB_1_ induces a mixture of DNA lesions that are repaired not only by NER but also by other pathways where SSB intermediates are less rapidly resolved. For example, a recent study shows that AFB_1_-Fapy-dG is partially repaired by BER (recognized and excised by the glycosylase NEIL1) in mammalian cells (103), and it has been shown that BER intermediates are readily detected, even without HU/AraC (104–106). Importantly, HU/AraC greatly increases the overall level of SSBs (Figure 3C), consistent with trapping NER intermediates. In the case of B[a]P treatment, there is a significant, but relatively small, increase in SSBs in the absence of HU/AraC, whereas the addition of HU/AraC reveals a remarkably strong dose–response to B[a]P (Figure 3D). Together, these results show that HU/AraC works well with primary mouse hepatocytes and can be used to enhance detection of DNA damage induced by chemicals that form bulky lesions following metabolic activation.

### Metabolic activation modulates the level of SSBs detected using HU/AraC approach

**Figure 4.**
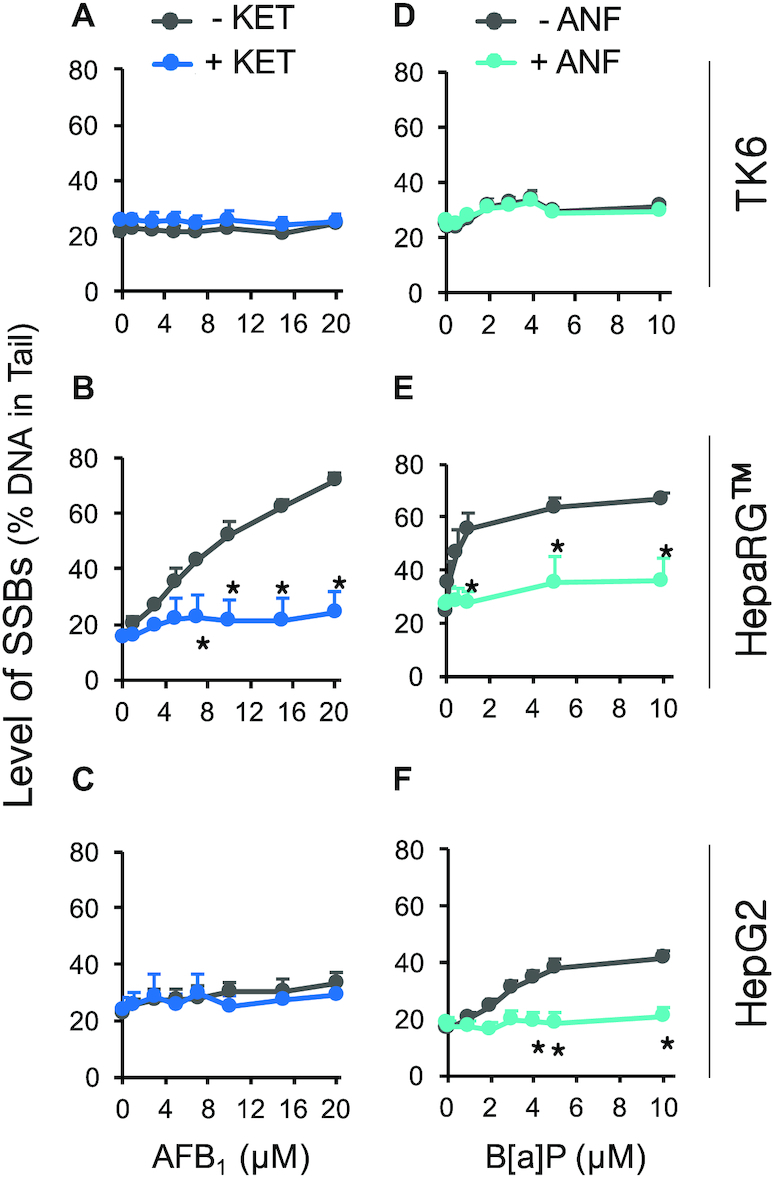
Role of metabolic activation in induction of SSBs by AFB_1_ and B[a]P. Cells were treated with AFB_1_ or B[a]P for 24 h in the presence of 1 mM HU and 10 μM AraC and analyzed with the alkaline CometChip. To inhibit AFB_1_ metabolic activation, 5 μM KET was added to AFB_1_ treatment (blue lines in (**A**), (**B**) and (**C**)). To inhibit B[a]P bioactivation, 25 μM ANF was added to B[a]P treatment (teal lines in (**D**), (**E**) and (**F**)). Gray lines represent treatment conditions without KET and ANF. (A) and (D) TK6 cells. (B) HepaRG™ cells (same-day treatment). (E) HepaRG™ (day-7 treatment). (C) and (F) HepG2 cells. *n*$ \ge$ 3. Error bars are standard error of the mean. * P < 0.05, two-way ANOVA with post hoc analysis by Bonferroni test.

To further validate that SSBs induced by AFB_1_ and B[a]P are due to metabolic activation of the carcinogens, we exploited CYP450 inhibitors. Specifically, we tested the possibility that inhibition of CYP450 enzymes would reduce the level of DNA adducts, which in turn would suppress the formation of NER-induced SSBs. To reduce metabolic activation of AFB_1_, we treated cells with ketoconazole (KET), a potent inhibitor of CYP3A4 (38,107–110). At 5 μM KET, CYP3A4 activity is reduced by ∼100-fold in HepaRG™ cells and by ∼10-fold in HepG2 cells (Supplemental Figure S3). As expected, KET reduces CYP3A4 activity regardless of HU/AraC (Supplemental Figure S3), and KET does not induce DNA damage in TK6, HepaRG™, or HepG2 cells (Figure 4A–C and Supplemental Figure S6 for untreated cells). When HepaRG™ cells are exposed to AFB_1_, there is a significant increase in the level of SSBs, as expected. However, in the presence of KET, the level of SSBs is reduced to near background levels (Figure 4B, Supplemental Table S3), indicating that AFB_1_ requires metabolic activation prior to formation of DNA damage. As expected, there is no significant increase in SSBs for TK6 or HepG2, and thus there is no impact of KET (Figures 3A, 4A, C and Supplemental Table S3). Together, these results show that HepaRG™ cells have the ability to metabolically activate AFB_1_ and that the vast majority of AFB_1_-induced damage is due to CYP3A4 activity.

B[a]P, like many other PAHs, is an inducer of the aryl hydrocarbon receptor (AhR) that regulates a number of phase I and phase II enzymes, including the CYP1 family (73). To inhibit the metabolism of B[a]P, we used α-naphthoflavone (ANF), which binds to AhR and inhibits its activation, thereby preventing the upregulation of the CYP1 family (73,111,112). In addition, ANF is also a potent antagonist of CYP1A2 (113,114). We observed that B[a]P induces CYP1A2 activity in HepaRG™ and HepG2 (Supplemental Figure S4, grey bars), consistent with activation of the AhR receptor. In the presence of 25 μM ANF, induction of CYP1A2 is prevented (Supplemental Figure S4), and ANF by itself does not induce DNA damage in TK6, HepaRG™, or HepG2 (Figure 4D–F and Supplemental Figure S6). Significantly, the same dose of ANF reduces the levels of B[a]P-induced SSBs to near background levels in both HepaRG™ and HepG2 (Figure 4E, F and Supplemental Table S3). These results show that, like AFB_1_, DNA damage induced by B[a]P is dependent on metabolic activation.

### Contribution of NER to SSB formation

**Figure 5.**
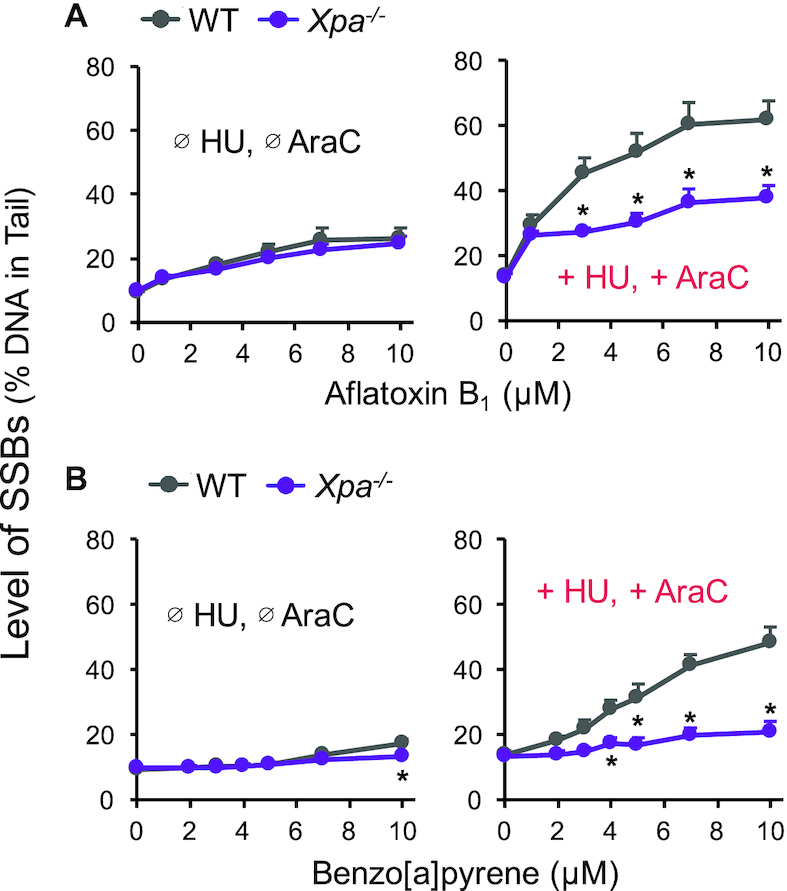
Application of *Xpa^−^^/^^−^* mouse hepatocytes to study the contribution of NER intermediates to SSBs detected by HU/AraC approach. Primary hepatocytes from six pairs of WT and *Xpa^−^^/^^−^* mice (C57Bl6, 10–14 weeks old) were isolated via two-step collagenase liver perfusion and incubated at 37°C overnight on CometChip (see Materials and Methods). Cells were then treated with AFB_1_ (**A**) or B[a]P (**B**) for 24 hours in the absence (Ø) or presence (+) of 1 mM HU and 10 μM AraC and analyzed with the alkaline CometChip. Each data point is the average of six mice. Error bars are standard error of the mean. *P < 0.05, two-way ANOVA with post hoc analysis by Bonferroni test.

Having shown that bulky lesions can be detected in primary mouse hepatocytes (Figure 3C and D), it is thus possible to exploit mouse models lacking key NER proteins. In order to directly test the role of NER in promoting SSBs, we used an *Xpa^−^^/^^−^* mouse model to completely abolish NER (115). The XPA protein is an essential component of the NER preincision complex, interacting with a number of NER proteins (e.g. TFIIH, RPA, ERCC1-XPF and PCNA) to enable incision (11). While there is a clear dose response in WT cells showing increased SSBs following exposure to AFB_1_ in the presence of HU/AraC (Figure 5A, right), in *Xpa^−^^/^^−^* cells, AFB_1_-induced SSBs are greatly reduced (Figure 5A, right). Similarly, B[a]P induces SSBs in WT cells, but not significantly in *Xpa^−^^/^^−^* cells (Figure 5B, right). These results indicate that NER intermediates contribute to most of the SSBs induced by AFB_1_ and B[a]P. Interestingly, for AFB_1_, even without HU/AraC, there is nevertheless a statistically significant increase in SSBs relative to untreated cells for both WT and *Xpa^−^^/^^−^* cells, albeit small in magnitude. This observation is consistent with the possibility that NER-independent enzymes contribute to AFB_1_-induced SSBs. Since it is known that AFB_1_ induces oxidative stress (116–118), and that oxidative lesions are repaired by BER it is possible that BER of oxidative lesions contributes to the low level of NER-independent SSBs.

### HepaCometChip is a sensitive assay for genotoxic agents

For performance assessment of *in vitro* genotoxicity tests, the European Reference Laboratory for Alternatives to Animal Testing (EURL ECVAM) published recommendations of chemicals that should give either positive results or negative results in an *in vitro* test (119,120). To assess the sensitivity of the HepaCometChip, we treated HepaRG™ cells with nine known *in vivo* genotoxic agents from Group 1 of the ECVAM’s recommendation (120) (Supplemental Table S2, No. 4–12) and compared the levels of SSBs in the absence and presence of HU/AraC. Remarkably, whereas only one chemical shows a positive result for DNA damage in the absence of HU/AraC (namely N-nitrosodimethylamine, or NDMA) (Table 1, third and fourth columns), the presence of the repair inhibitors reveals significant DNA damage for seven (out of nine) known positive compounds (Table 1, fifth and sixth columns). Thus, inhibition of NER completion converts seven false negatives (in the absence of HU/AraC) into correct positives (using HU/AraC). In addition, four of the test compounds are known to be metabolically activated [cyclophosphamide, B[a]P, NDMA, and 2,4-diaminotoluene (2,4-DAT) (120)], and all four are scored as positives using HU/AraC with the HepaRG™ cells. Among the seven chemicals that were scored as positives for DNA damage in the presence of HU/AraC, high levels of cytotoxicity were observed for hydroquinone (HQ) and chloramphenicol (CAM) (Supplemental Figure S7F and H). Although there is no formal threshold for cell viability in scoring the comet assay, cytotoxicity may contribute to DNA fragmentation, which can lead to overestimation of genotoxicity (79). If a 50% cell viability threshold is applied, then HQ and the top dose of CAM will be excluded from the positive results. In the case of etoposide, while statistical significance is only achieved with HU/AraC, the dose response trends appear to be similar in both the absence and presence of HU/AraC (Supplemental Figure S7A).

**Table 1.** Comparisons among the Ames assay, traditional alkaline comet assay conditions, and conditions that trap NER intermediates (+ HU + AraC) in a genotoxic screen of nine known *in vivo* genotoxins (120). Statistical significance is designated as ‘+’ and ‘−’. Dose ranges for statistically significant results are indicated

	Genotoxicity					
		Alkaline CometChip HepaRG™		Alkaline CometChip HepaRG™ (+ HU + AraC)		Carcinogenicity
*In vivo* genotoxins	Ames	+/−	‘+’ dose range	+/−	‘+’ dose range	IARC classification (92)
Etoposide	+ (134)	−		+	10 μM	Group 1
2,4-DAT	+ (135)	−		+	10 mM	Group 2B
CP	+ (135)	−		+	5–10 mM	Group 1
PCA	+ (135)	−		−		Group 2B
NDMA	+ (135)	+	2.5 - 20 mM	+	2.5 - 20 mM	Group 2A
HQ	− (135)	−		+	0.33 mM	Group 3
B[a]P	+ (135)	−		+	5–10 μM	Group 1
CAM	− (136)	−		+	3.1 mM	Group 2A
Cisplatin	+ (137)	−		−		Group 2A

IARC classification: Group 1: human carcinogen, Group 2A: probably human carcinogen, Group 2B: possible human carcinogen, Group 3: not classifiable as to its carcinogenicity to humans.

The two compounds that showed negative results are *p-*chloroaniline (PCA) and cisplatin. PCA is used in a number of industrial processes, such as dye production. Although PCA is an *in vivo* genotoxic agent (119,120), a rodent carcinogen, and a possible human carcinogen (Table 1), there are conflicting data about PCA’s DNA damaging potential (WHO CICAD report 2003, https://www.who.int/ipcs/publications/cicad/en/cicad48.pdf). Notably, although the highest dose (5 mM) induces ∼50% cell death, there is nevertheless no significant increase in DNA damage (Supplemental Figure S7D). These results suggest that the *in vivo* genotoxic potential of PCA depends on processes that are independent from formation of DNA damage. Cisplatin was also negative. Cisplatin is a commonly used platinum-based chemotherapeutic for many cancers, including bladder, ovarian, head and neck, and non-small-cell lung cancer (121–124). Upon entering the cell, the chloride ligands hydrolyze, generating aquated cisplatin that can bind to the N7 atom of purine bases to form intra- and interstrand crosslinks (121). It is well established that crosslinks inhibit DNA migration in the comet assay, therefore a negative result for cisplatin is expected based on its mechanism of action (125).

In the absence of HU/AraC, only NDMA shows positive results for DNA damage (Table 1, third and fourth columns). NDMA is bioactivated in the body mainly by CYP2E1 (126) to yield an α-hydroxymethyl nitrosamine that forms a reactive methyl diazonium ion, which methylates nucleobases via S_N_1 nucleophilic substitution (WHO CICAD report 2002, https://www.who.int/ipcs/publications/cicad/en/cicad38.pdf). Methylated bases are repaired primarily by the base excision repair (BER) pathway. As BER intermediates (including abasic sites and SSBs) are readily detected by the alkaline comet assay, the observation that NDMA yields a positive result in the absence of HU/AraC is therefore expected.

As another example of how the combination HU/AraC can be applied to study genotoxicity, we tested the DNA damaging potential of a classic antimalarial therapeutic, artesunate (127). We found that HU/AraC reveals a wider range of genotoxic artesunate doses compared to the traditional assay (Supplemental Figure S8). The results are consistent with previous studies showing that artesunate causes DNA damage in mammalian cells (127).

Taken together, the repair synthesis inhibitors HU and AraC significantly improve and extend the sensitivity of the alkaline comet assay. In the context of chemical genotoxicity testing, we propose the use of the alkaline CometChip with HepaRG™ cells in the presence of HU/AraC (HepaCometChip), as a screening platform to achieve high throughput and significantly decreased false negative rates.

## DISCUSSION

Despite their known carcinogenicity, to date, no high throughput methods had been developed for detecting bulky DNA lesions. Here, we have leveraged the comet assay to overcome this limitation, thus opening doors to improved detection of potential carcinogens. To increase the broad sensitivity of the alkaline comet assay, we combined the metabolic capacity of hepatic cells with small molecule inhibitors of NER repair synthesis so that bulky lesions can be formed and, through aborted repair, converted into detectable SSBs. We also performed the comet assay using the CometChip, thus achieving far greater throughput and sensitivity (15,16). The HepaCometChip enables rapid and sensitive detection of DNA damaging agents that create bulky lesions.

Here, we studied three carcinogenic DNA damaging agents known to create bulky lesions, namely UV-C, AFB_1_ and B[a]P. To specifically test whether the SSBs are the result of NER activity, we used cells that were completely lacking key enzymes required for NER initiation. In the absence of XPG, UV-induced SSBs are virtually abolished. Further, primary mouse hepatocytes lacking Xpa similarly showed a dramatic reduction in SSBs induced by AFB_1_ and B[a]P. Together, these results show definitively that bulky lesions can be detected by formation of downstream NER intermediates.

A significant barrier to the detection of DNA damaging agents is the frequent requirement for metabolic activation. Many pro-carcinogens are converted into DNA reactive metabolites by CYP450s. Nevertheless, most current genotoxicity screens are performed with cell types that do not support metabolic activation, leading to a blind spot when screening for potential carcinogens. To overcome this limitation, we incorporated hepatic cells into the platform. When HepaRG™ and HepG2 were treated with either AFB_1_ or B[a]P, we observed a significant increase in NER intermediates, in sharp contrast to TK6 cells that are not capable of metabolic activation. To formally test the approach of harnessing metabolism to convert AFB_1_ and B[a]P into chemicals that can damage DNA, we used known inhibitors of CYP450 activity. Specifically, using CYP450 inhibitors in HepaRG™ and HepG2 cells, we verified that formation of SSBs upon AFB_1_ and B[a]P treatments is dependent on the activity of CYP3A4 and CYP1A2, respectively. Taken together, these results show that our HepaCometChip platform captures relevant CYP450 activities that are required for detecting metabolically activated DNA damaging agents. Interestingly, these results also point to the ability to use the HepaCometChip as a means for probing the specific roles of CYP450s in inducing genotoxicity. As a potential application for novel compounds with unknown metabolism, the HepaCometChip platform can be used to screen a panel of CYP450 inhibitors to differentiate between parent- and metabolite-based genotoxicity and to determine the contribution of specific CYP450s.

In these studies, we also compared HepaRG™ and HepG2 cells for their efficacy in detecting bulky lesions. With its broad-spectrum metabolism and its high basal and inducible metabolic enzyme levels, HepaRG™ has the potential to be a robust and reliable cell model for genotoxicity testing. We found that HepaRG™ exhibit orders of magnitude higher activity levels of CYP3A4 and CYP1A2, consistent with the difference in gene expression levels found in other studies (91,92,97). Importantly, we also observed that the higher CYP3A4 and CYP1A2 activities in HepaRG™ cells translate to higher levels of DNA damage induced by AFB_1_ and B[a]P compared to HepG2. Furthermore, a recent study utilized both HepaRG™ and HepG2 cells on CometChip to test genotoxicity of a variety of chemicals with varying metabolic capacity and found that CometChip assay on HepaRG™ cells was more effective in detecting genotoxic carcinogens requiring metabolic activation (128). Together, these results point to the use of HepaRG™ cells on the CometChip as an effective strategy for broad detection of metabolically activated DNA damaging agents.

Primary hepatocytes are a critical tool in toxicity testing and metabolism studies. Here, we investigated the efficacy of studying primary hepatocytes using the CometChip. We demonstrated that immediately after isolation, primary mouse hepatocytes can be easily loaded onto the CometChip, exposed to genotoxic agents and analyzed for SSBs directly on chip. Published methods for studies of primary hepatocytes often involve two-dimensional culturing on tissue culture dishes (102). By loading directly onto the CometChip, analysis of primary hepatocytes is simplified and eliminates the need to detach the cells from culturing vessels (usually via trypsinization), thus minimizing the stress on the cells. We demonstrated here that the hepatocytes on CometChip maintain their ability to metabolically activate the carcinogens AFB_1_ and B[a]P, indicating that CometChip can potentially be used as a suitable culturing platform for primary hepatocytes. Because CometChip is fabricated with agarose, which provides a hydrophilic and neutrally charged surface, we expect that culturing primary hepatocytes on-chip will yield similar results compared to ultra-low attachment plates (e.g. Corning^®^ Ultra-Low Attachment Spheroid Microplates) that are routinely used for hepatocyte spheroid formation and culture (129). Given the efficacy of this approach for studies of mouse hepatocytes, we anticipate that these methods would be equally effective for future studies of primary human hepatocytes.

Although we have shown that we can leverage NER intermediates as an indicator of bulky lesions, it remains possible that HU/AraC may also increase sensitivity for the detection of lesions repaired by other repair pathways (such as BER). Intriguingly, while HU/AraC is exploited here as a way to trap NER intermediates, ostensibly to convert undetectable bulky lesions into detectable single strand breaks, the concept of revealing base lesions via conversion to strand breaks is not new. Extensive work has been done to exploit purified glycosylases as a tool for expanding the sensitivity of the comet assay. To accomplish this, after lysis, the DNA of pre-comets is incubated with glycosylases that convert undetectable base lesions into single strand breaks (130,131). As one example, Fpg has been used extensively to convert its substrates (including 8-oxoguanine) into detectable strand breaks for comet analysis (131–133). Unlike BER (wherein damaged bases can be converted to strand breaks using a single purified bifunctional glycosylase), many proteins need to be present in order for NER to cleave the backbone near the site of the lesion, making *in vitro* studies complex. For this reason, in this work, we have focused on exploiting NER capacity that is inherent to live cells as a way to reveal bulky lesions.

As a screening tool for genotoxicity, having a platform that detects a broad range of DNA damaging agents is a great asset. In fact, to test the efficacy of the HepaCometChip platform for screening potential carcinogens, we compared the traditional comet assay (without HU/AraC) to the HepaCometChip for nine known genotoxic agents. A positive result was observed for seven agents using the HepaCometChip, all of which were missed using the traditional comet assay. PCA is a known genotoxic agent *in vivo*, but its mechanism of action is not well understood. The observation that PCA is negative on the HepaCometChip suggests that it may be an indirect acting genotoxic agent, e.g. one that does not directly damage DNA. The other genotoxin negative on the HepaCometChip was cisplatin, which forms interstrand crosslinks. It is well established that crosslinks inhibit DNA migration. Therefore, the negative result for cisplatin is consistent with its mode of action. Taken together, the combination of leveraging hepatocyte metabolism and DNA repair synthesis inhibition provides a highly sensitive approach for detecting DNA damaging agents that show a false negative result using the traditional comet assay.

With regard to limitations, the HepaCometChip is not as sensitive as assays that detect specific DNA lesions for which there is prior knowledge of adduct structures, such as HPLC and mass spectrometry. However, for applications where the structure is not known in advance of the assay, the HepaCometChip is preferable. In addition, for primary screens, it is generally the case that relatively high doses of an agent can be tested, making sensitivity less important. Nevertheless, for some small molecule libraries where compound quantities are limited, the amount of compound available could lead to formation of adducts that are below the level of detection. Finally, one other limitation is that HU/AraC, while effective for trapping NER intermediates, might potentially also trap intermediates formed during BER. This lack of specificity could be considered to be a strength, however, since as a screening tool, having a broad sensitivity can be advantageous. Further, for experiments where it is important to know if a DNA adduct is repaired specifically via NER, cell lines deficient in essential NER components could be useful as a means for determination of NER’s contribution.

In conclusion, using a combination of the DNA repair synthesis inhibitors HU and AraC and a metabolically competent human cell line HepaRG™, we developed a CometChip platform for HT genotoxicity testing that has exquisite sensitivity for bulky DNA adducts. The platform can be used as a powerful HT tool for screening large chemical libraries, with applications in safety testing for both public health and the pharmaceutical industry. The use of HU/AraC together with CometChip is also a promising tool for clinical applications, where DNA damage levels can be monitored as a surrogate endpoint for tumor response. Taken together, the HepaCometChip fills a gap in genotoxicity testing by capturing agents that are negative using traditional comet analysis, and as such will serve as a useful tool for a broad range of applications.

## Supplementary Material

gkz1077_Supplemental_File

## Notes

Present address: Le P. Ngo, Department of Biological Engineering, Massachusetts Institute of Technology, Cambridge, MA 02139, USA.

Present address: Norah A. Owiti, Department of Biological Engineering, Massachusetts Institute of Technology, Cambridge, MA 02139, USA.

Present address: Carol Swartz, Toxicology Program, Integrated Laboratory Systems, Inc., Research Triangle Park, NC 27560, USA.

Present address: John Winters, Toxicology Program, Integrated Laboratory Systems, Inc., Research Triangle Park, NC 27560, USA.

Present address: Yang Su, Department of Biology, Massachusetts Institute of Technology, Cambridge, MA 02139, USA.

Present address: Jing Ge, Navigant Consulting, Boston, MA 02110, USA.

Present address: Aoli Xiong, BioSystems and Micromechanics, Singapore-MIT Alliance for Research and Technology, 138602 Singapore.

Present address: Jongyoon Han, Department of Biological Engineering and Department of Electrical Engineering, Massachusetts Institute of Technology, Cambridge, MA 02139, USA.

Present address: Leslie Recio, Toxicology Program, Integrated Laboratory Systems, Inc., Research Triangle Park, NC 27560, USA.

Present address: Leona D. Samson, Department of Biological Engineering and Department of Biology, Massachusetts Institute of Technology, Cambridge, MA 02139, USA.

Present address: Bevin P. Engelward, Department of Biological Engineering, Massachusetts Institute of Technology, Cambridge, MA 02139, USA.
