# Supplementary material for: Sensitive CometChip assay for screening potentially carcinogenic DNA adducts by trapping DNA repair intermediates
Source: Nucleic Acids Res. 2019 Dec 11;48(3):e13. doi: 10.1093/nar/gkz1077 (PMC7026589; doi:10.1093/nar/gkz1077)
Supplement: gkz1077_Supplemental_File [file gkz1077_supplemental_file.pdf]

Nucleic Acids Research - Supporting Information

Sensitive CometChip Assay for Screening Potentially Carcinogenic DNA Adducts by Trapping DNA Repair Intermediates

Le P. Ngo[1], Norah A. Owiti[1], Carol Swartz[2], John Winters[2], Yang Su[3], Jing Ge[1], Aoli Xiong[4], Jongyoon Han[1, 5], Leslie Recio[2], Leona D. Samson[1,3], and Bevin P. Engelward[1*]

[1] Department of Biological Engineering, Massachusetts Institute of Technology, Cambridge, MA, 02139, USA
[2] Toxicology Program, Integrated Laboratory Systems, Inc., Research Triangle Park, NC, 27560, USA
[3] Department of Biology, Massachusetts Institute of Technology, Cambridge, MA, 02139, USA
[4] BioSystems and Micromechanics, Singapore-MIT Alliance for Research and Technology, 138602, Singapore
[5] Department of Electrical Engineering, Massachusetts Institute of Technology, Cambridge, MA, 02139, USA

* To whom correspondence should be addressed. Tel: (+1) 617-258-0260; Fax: (+1) 617-258-0499; Email: bevin@mit.edu

Table S1

List of important abbreviations.

Abbreviation	Definition
SSB	Single Strand Break
BER	Base Excision Repair
NER	Nucleotide Excision Repair
XPG	Xeroderma Pigmentosum Complementation Group G protein
XPA	Xeroderma Pigmentosum Complementation Group A protein
HU	Hydroxyurea
AraC	1-β-D-arabinofuranosyl cytosine
PAHs	Polycyclic aromatic hydrocarbons
B[a]P	Benzo[a]pyrene
AFB_1	Aflatoxin B_1
NDMA	N-nitrosodimethylamine
GSH	L-Glutathione
KET	Ketoconazole
ANF	α-naphthoflavone
CYP450s	Cytochrome CYP450 enzymes

Table S2

List of chemicals used in this study that were prepared from powder form.

No.	Chemical	Abbreviation	Catalog number	Stock concentration	Solvent
1	Aflatoxin B_1	AFB_1	AF10	4 mM	DMSO
2	Ketoconazole	KET	K1003	20 mM	DMSO
3	α-Naphthoflavone	ANF	N5757	20 mM	DMSO
4	Benzo[a]pyrene	B[a]P	B1760	20 mM and 1 mM	DMSO
5	Etoposide		E1383	1 mM	DMSO
6	2,4-Diaminotoluene	2,4-DAT	D0123	1 M	DMSO
7	Cyclophosphamide monohydrate	CP	C0768	1 M	DMSO
8	*p*-*C*hloroaniline	PCA	C22415	500 mM	DMSO
9	N-Nitrosodimethylamine	NDMA	48552	1 M	H_2O
10	Hydroquinone	HQ	H17902	100 mM	H_2O
11	Chloramphenicol	CAM	C0378	200 mg/ml (619 mM)	DMSO
12	cis-Diamineplatinum(II) dichloride	cisplatin	479306	1 mM	H_2O
13	Artesunate		A3731	50 mM	7.5% $NaHCO_3$ solution

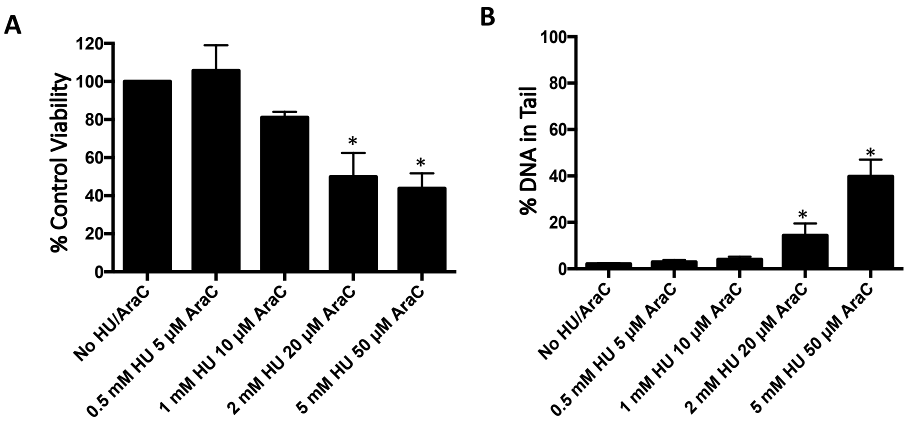

Figure S1. Effects of HU/AraC on HepaRG cell viability and DNA damage. HepaRG cells were incubated with indicated doses of HU/AraC and analyzed for cytotoxicity using Trypan Blue exclusion test **(A),** and for DNA damage using Alkaline CometChip **(B),** 24 hours following exposure. * $p < 0.05$, one-way ANOVA with Post hoc analysis by Dunnett's multiple comparison test (between each HU/AraC dose and the untreated control). $n \geq 3$. Error bars are standard error of the mean.

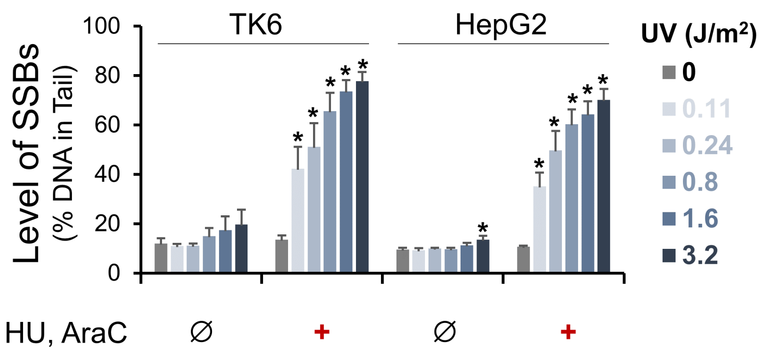

Figure S2. HU/AraC approach reveals dose-response to UV exposure. TK6 and HepG2 cells were irradiated with indicated doses of UV-C and analyzed for SSBs one hour following exposure. Cells were either incubated with the repair synthesis inhibitors (1 mM HU, 10 μM AraC) for 40 minutes prior to UV irradiation and one hour of repair after exposure (+) or were incubated in regular medium without the inhibitors (Ø). * $p < 0.05$, one-way ANOVA with Post hoc analysis by Dunnett's multiple comparison test (between each UV dose and the untreated control). n ≥ 3. Error bars are standard error of the mean.

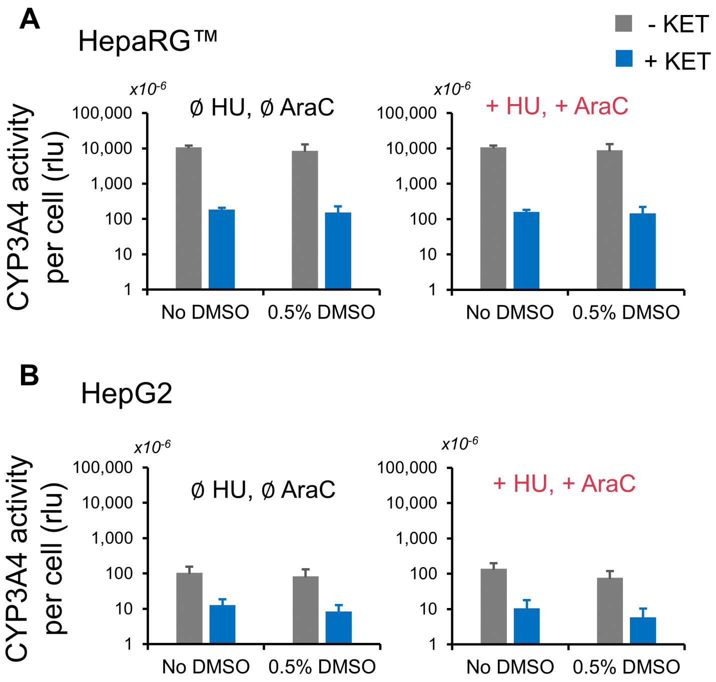

Figure S3. Effects of ketoconazole (KET) on CYP3A4 activity per cell measured by CYP450-Glo™ assay. Cells were incubated with 5 μM KET for 24 hours at 37°C in the absence (Ø) or presence (+) of 1 mM HU and 10 μM AraC. Gray bars: basal CYP3A4 activity levels for both untreated (No DMSO) and vehicle control (0.5% DMSO). Blue bars: CYP3A4 activity levels in the presence of 5 μM KET. (**A**) HepaRG™ cells. (**B**) HepG2 cells. rlu: relative light unit. n ≥ 3. Error bars are standard error of the mean.

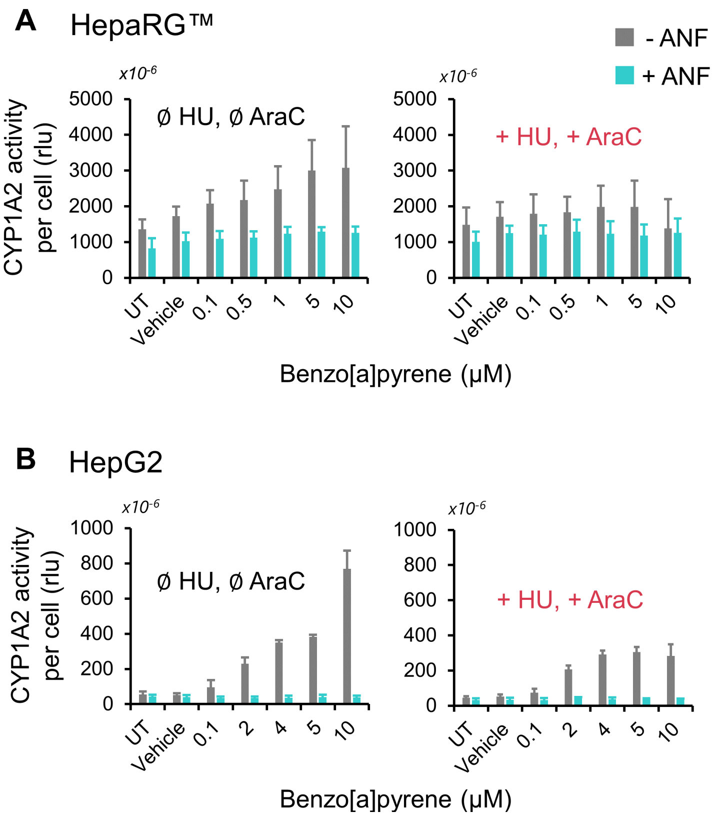

Figure S4. Effects of α-naphthoflavone (ANF) on CYP1A2 activity per cell measured by CYP450-Glo™ assay. Cells were incubated with B[a]P together with 25 µM ANF for 24 hours at 37°C in the absence (Ø) or presence (+) of 1 mM HU and 10 µM AraC. Gray bars: CYP1A2 activity levels corresponding to untreated control (UT), vehicle control (0.5% DMSO), and B[a]P doses. Teal bars: CYP1A2 activity levels in the presence of 25 µM ANF. (**A**) HepaRG™ cells. (**B**) HepG2 cells. rlu: relative light unit. n ≥ 3. Error bars are standard error of the mean.

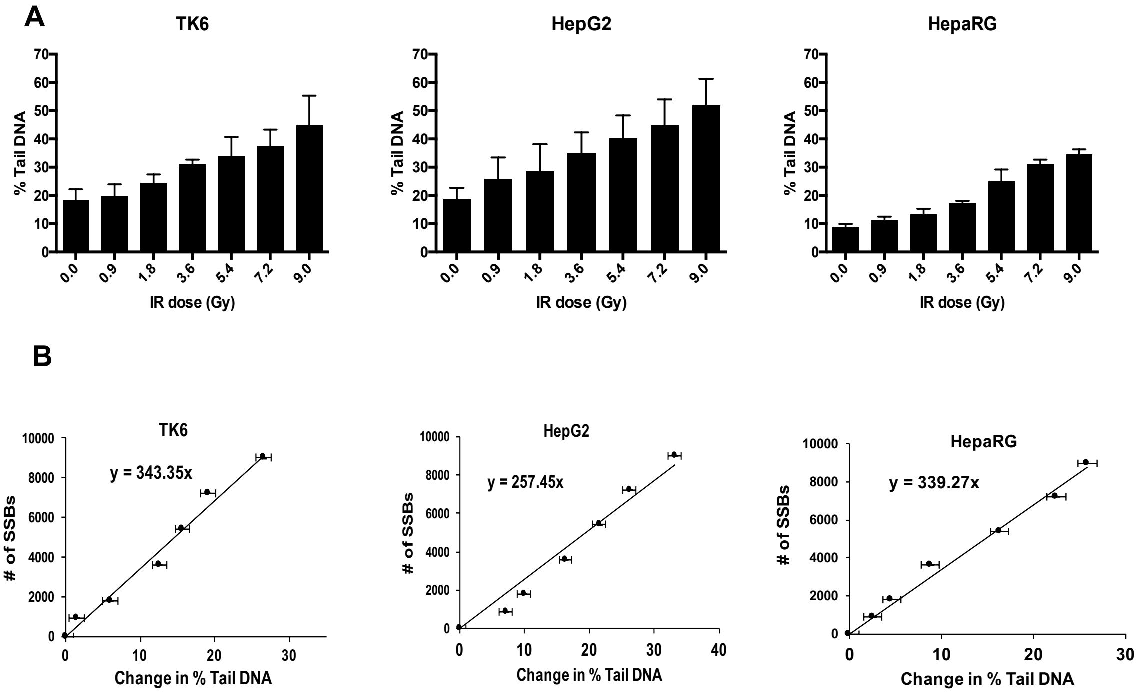

Figure S5. Level of DNA damage and calibration curves following γIR. TK6, HepG2 and HepaRG cells were irradiated with γIR at the indicated doses and analyzed with alkaline CometChip **(A).** To correlate the SSBs induced with the gamma radiation dose, the percent tail DNA from non-radiated cells were subtracted from the % Tail DNA for each dose and a linear calibration curve generated **(B)**. The slopes of the curves were used to estimate the number of SSBs/cell induced following each chemical treatment (data shown in Table S3)

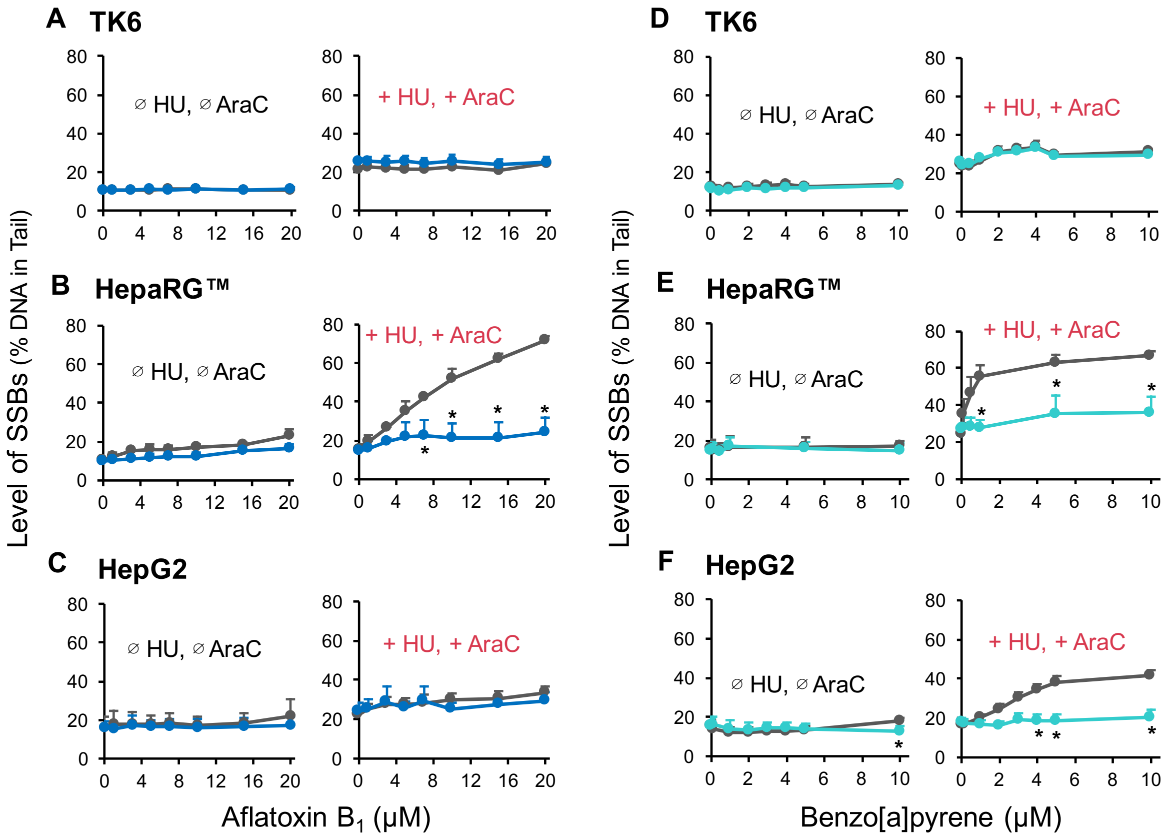

Figure S6. Role of metabolic activation in induction of SSBs by AFB_1 and B[a]P (same dataset as **Fig. 4** but also including ∅ HU, ∅ AraC controls). Cells were treated with AFB_1 or B[a]P for 24 hours in the absence (Ø) or presence (+) of 1 mM HU and 10 μM AraC and analyzed with the alkaline CometChip. To inhibit AFB_1 metabolic activation, 5 μM KET was added to AFB_1 treatment (blue lines in (**A**), (**B**), and (**C**)). To inhibit B[a]P bioactivaion, 25 μM ANF was added to B[a]P treatment (teal lines in (**D**), (**E**), and (**F**)). Gray lines represent treatment conditions without KET and ANF. (**A**) and (**D**) TK6 cells. (**B**) HepaRG™ cells (same-day treatment). (**E**) HepaRG™ (day-7 treatment). (**C**) and (**F**) HepG2 cells. n ≥ 3. Error bars are standard error of the mean. * $p < 0.05$, two-way ANOVA with Post hoc analysis by Bonferroni test.

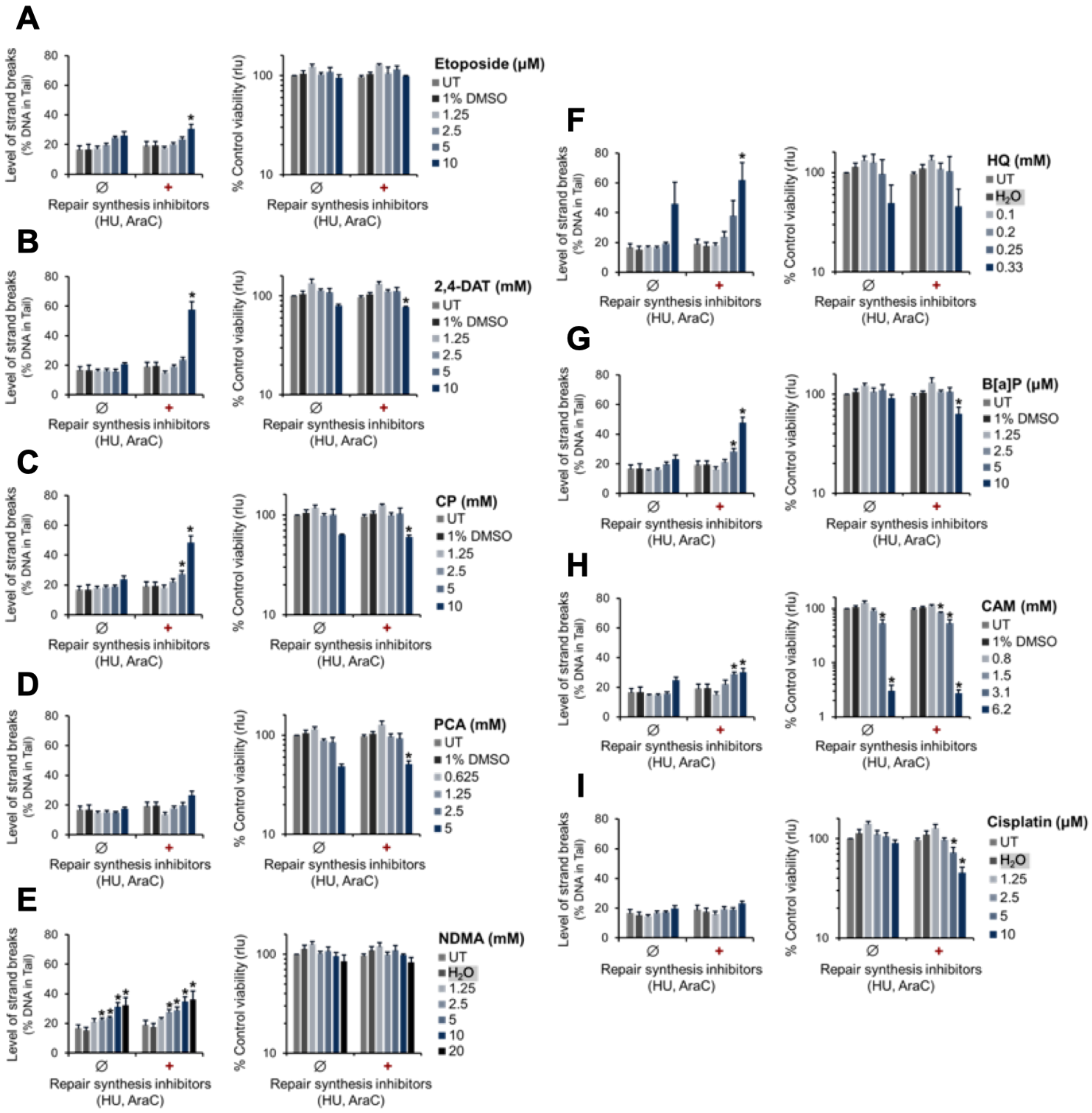

Figure S7. DNA damage in HepaRG™ cells induced by nine *in vivo* genotoxins, measured by the alkaline CometChip using the repair synthesis inhibitors HU and AraC. HepaRG™ cells were exposed to test compounds for 24 hours at 37°C using the same-day treatment procedure (see **Methods**). 1 mM HU and 10 μM AraC were either absent (Ø) or present (+) in the same treatment. At the end of the exposure period, half of the cells were analyzed for SSB levels with the alkaline CometChip (left plot of each figure), and the other half were analyzed for cell viability using the CTG® assay (see **Methods**) (right plot of each figure). "UT" represents background damage. Vehicle controls are "1% DMSO" or "H_2O". (**A**) Etoposide. (**B**) 2,4-Diaminotoluene. (**C**) Cyclophosphamide. (**D**) pAraChloroaniline. (**E**) N-Nitrosodimethylamine. (**F**) Hydroquinone. (**G**) Benzo[a]pyrene. (**H**) Chloramphenicol. (**I**) cis-Diamineplatinum (II) dichloride. rlu: relative light unit. $n \geq 3$. Error bars are standard error of the mean. * $p < 0.05$, one-way ANOVA with Post hoc analysis by Dunnett's multiple comparison test (between a treatment dose and the corresponding vehicle control).

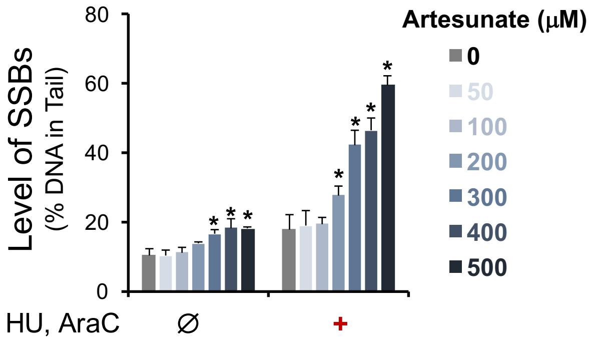

Figure S8. DNA damage induced by antimalarial agent artesunate in HepG2 cells. HepG2 cells were exposed to artesunate for 24 hours in the absence (∅) or presence (+) of 1 mM HU and 10 μM AraC. "0" represents the vehicle control, 0.07% sodium bicarbonate. n ≥ 3. Error bars are standard error of the mean. * $p < 0.05$, one-way ANOVA with Post hoc analysis by Dunnett's multiple comparison test (between treated and untreated control).

Table S3

Quantitation of single strand breaks following B[a]P and AFB_1 treatments.

Cell Line	Chemicals	Average % Tail DNA		Approximate SSBs induced by chemicals (adjusting background)*	
		-HU/AraC	+HU/AraC	-HU/AraC	+HU/AraC
HepaRG	Untreated	17.2	28.9	0	0
	20 μM AFB_1	23.1	72.1	4,000	19,000
	20 μM AFB_1 +KET	16.7	24.3	1,800	3,000
	50 μM B[a]P	24.6	68.1	2,500	13,000
	10 μM B[a]P	16.9	66.8	-117	12,800
	10 μM B[a]P + ANF	14.5	36	-933	2,300
HepG2	Untreated	17.1	19.5	0	0
	20 μM AFB_1	21.9	33.4	1,200	3,500
	20 μM AFB_1 +KET	17	29.3	300	2,100
	50 μM B[a]P	19.4	40.4	600	5,400
	10 μM B[a]P	18.22	41.7	200	5,700
	10 μM B[a]P + ANF	12.7	20.7	-355	100
TK6	Untreated	11.9	20.7	0	0
	20 μM AFB_1	10.6	24.4	-463	1,200
	20 μM AFB_1 +KET	11.1	25.7	160	-13.7
	50 μM B[a]P	13.3	25.1	460	1,500
	10 μM B[a]P	12	26.1	30	1,800
	10 μM B[a]P + ANF	10.9	25	70	1,800

*Estimation of the number of SSBs induced by B[a]P and $AFB_{1,}$ calculated assuming 1Gy of gamma radiation induces 1000 SSBs.
